# The Pavlovian interpretation of speech and aphasia: Alexander Luria and Wilder Penfield

**DOI:** 10.3389/fpsyg.2024.1404953

**Published:** 2024-07-23

**Authors:** Richard Leblanc

**Affiliations:** Montreal Neurological Institute and McGill University, Montréal, QC, Canada

**Keywords:** aphasia, conditioned reflex, Luria, Pavlov, Penfield, speech

## Abstract

This paper discusses a neglected aspect of the historiography of aphasia, the role that Pavlovian conditioning played in Alexander Luria’s and Wilder Penfield’s understanding of the acquisition, expression, and loss of spoken and written speech. Luria was born into a bourgeois family in Tzarist Russia and pursued his research on speech and aphasia under the Soviet regime. Luria’s work was condemned in the last years of Stalin’s rule, but it received international acclaim in the West after Stalin’s death. Penfield was conversant with Pavlov’s writing having had a working relationship with one of Pavlov’s foremost students, Boris Babkin, who came to McGill University and later to the Montreal Neurological Institute after being jailed and exiled from the Soviet Union for lack of revolutionary fervor. Both Luria and Penfield, the latter as early as 1935, saw in Pavlovian conditioning mediated by specific areas of the human cerebral cortex the basic neurophysiological mechanism underlying speech and thought, and in Penfield’s’ case, memory, perception, self-awareness, and purposeful behavior. It is concluded that Luria and Penfield independently arrived at a general hypothesis, based on Pavlovian conditioning, that united the localization of speech, the syndromes caused by damage to speech-competent regions, and the putative neurophysiological mechanisms that they believed to underlie speech and higher cortical functions.

## Introduction

*It is nothing other than words which have made us human*—Pavlov^1^

In the spring of 1924, Ivan Pavlov gave a series of 23 lectures at the Military Medical Academy in Petrograd, now Saint Petersburg, that he gathered under the tittle *Conditioned Reflexes. An investigation of the Physiological Activity of the Cerebral Cortex*, which were translated into English in 1927. At the very beginning of his first lecture, *The development of the objective method in investigating the physiological activities of the cerebral hemispheres*, Pavlov admonished his contemporaries for not having taken further the work of Hitzig and Ferrier localizing the motor cortex, stating, “the important question of the physiologic mechanism of the whole higher and complex behavior of the animal (which is), dependent upon the cerebral hemispheres, has got hidden away in a corner, and this unlimited field, so fertile in possibilities for research, has never been adequately explored.” This was so, Pavlov continued, because “the activities of the hemispheres have been talked about as some kind of special psychical activity, [which] happens to have been annexed to the special field of another science--psychology,” and bemoaned that this had hampered the experimental study of the neurophysiological mechanisms underlying cortical function, including human speech (Pavlov, 1927, 2–3).

The Russian psychologist Alexander Rabinovitch Luria relied on Marxist theory and on the establishment of conditioned reflexes within functionally distinct cortical areas of the left hemisphere to explain the interpretation and expression of speech. Aphasic syndromes arose from interruption of the conditioned reflex arcs within these language competent areas. Although not as explicitly as Luria, Penfield independently proposed that Pavlovian conditioning underlies speech and other cognitive functions (Leblanc, 2019a,b,c).

**Figure 1:**
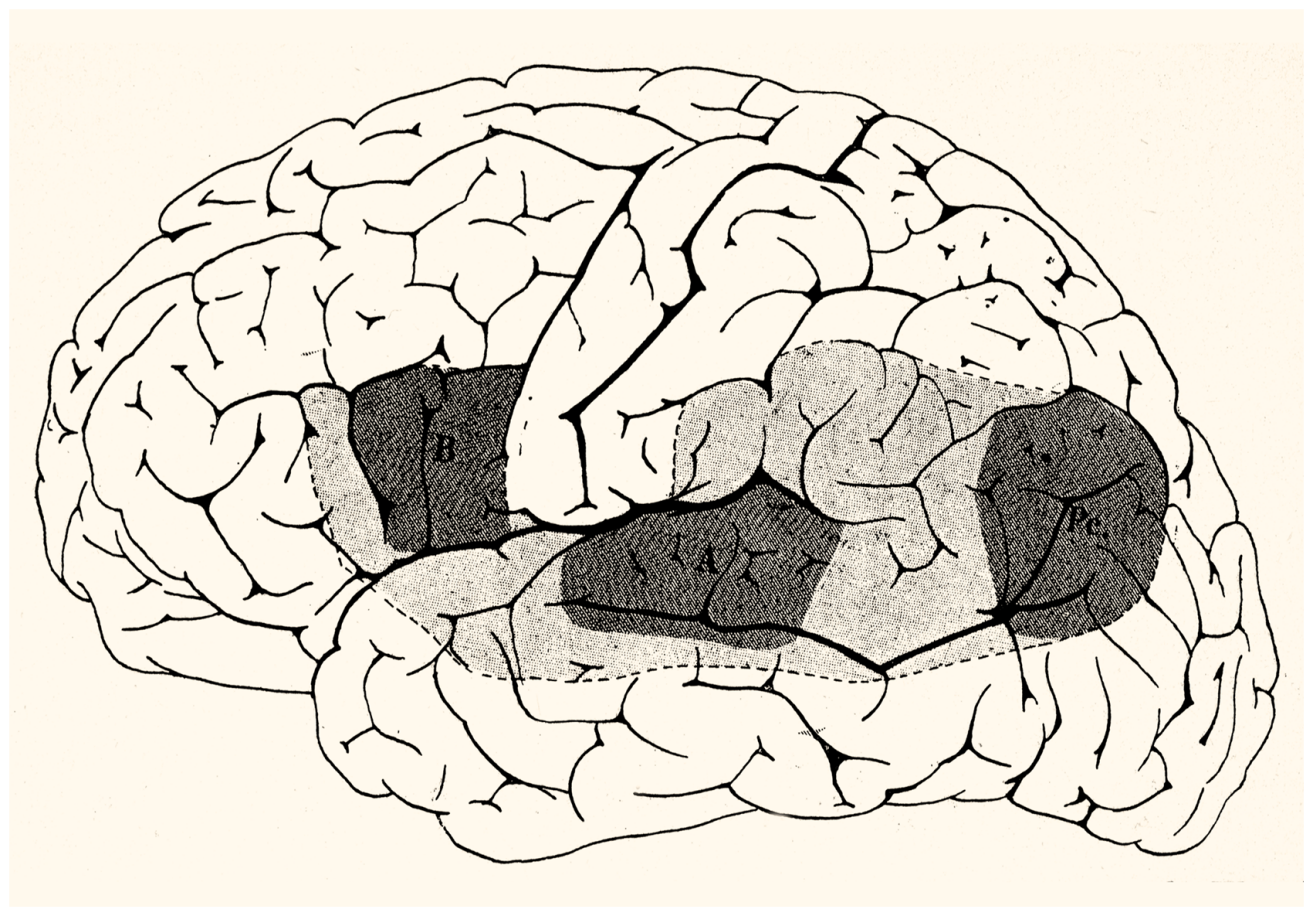
Dejerine’s “language zone.” Legend: B, Broca’s area; A, Wernicke’s area; Pc, pli courbe – the angular gyrus. The intensity of the shading reflects the probability of finding a speech deficit, articulate, verbally receptive, or of written language, particular to each area. A lesion in any part of the shaded area, or of the subcortical fibres joining them, can affect all aspects of language, but the aspect most affected will be determined by the proximity of the lesion to area A, B, or Pc (Public domain, Dejerine and Dejerine-Klumpke, 1901).

The first part of this paper addresses the Pavlovian theory of speech and aphasia as formulated by Luria in his study of the effects of traumatic cortical lesions sustained by Soviet soldiers during the Great Patriotic War (1941–1945), and how this led him to expand the speech-competent areas of the left hemisphere to include, beyond the areas described by Broca, Wernicke, and Dejerine (Figure 1), the premotor region and the border zone of the temporal and parietal lobes with the occipital lobe. Although Luria concluded that this broad localization indicated that speech is an integrated function, damage to specific areas produced distinctive aphasic syndromes.

The second part of the paper addresses Wilder Penfield’s introduction to Pavlovian physiology through his friendship with Boris Babkin, one of Pavlov’s earliest collaborators, and his reading of the first English translation of Pavlov’s papers on the conditioned reflex and the cerebral cortex. Unlike Luria, whose findings relied solely on lesion analysis, Penfield performed electrocortical stimulation in awake patients as they performed language tasks to delineate areas where stimulation interfered with speech. Unlike Luria, however, Penfield found that the speech-competent cortex, with the addition of the supplementary motor area, is not as widespread over the left hemisphere as Luria contended.

The third part of the paper addresses Penfield’s reliance on the conditioned reflex to explain the acquisition of speech, memory, perception, and will.

It is concluded that Pavlovian conditioning was fundamental to Luria’s and Penfield’s understanding of speech, and, in Penfield’s case, of other cognitive functions commonly attributed to mind.

## Materials and methods

Luria’s papers and books on speech and aphasia and on higher mental functions published in English, and Penfield’s papers and books, as well as those of his research fellows on the same topics, have been reviewed for this essay.

By Broca’s area is meant the posterior third of the third, or inferior, left frontal convolution. It is abbreviated F-3 and is composed of the *pars opercularis* (Brodmann area 44) and the *pars triangularis* (Brodmann area 45). Broca’s area and the frontal cortex in front of the precentral – motor – gyrus is referred to as the premotor region and includes the remainder of F-3 and the first and second frontal convolutions, abbreviated F-1 and F-2, respectively. The supplementary motor area is at the posterior and medial aspect of F-1 in front of the leg and foot representation of the precentral gyrus. It is referred to as the SMA.

By Wernicke’s *area* is meant the posterior aspect of the first, or superior, left temporal convolution (Brodmann area 22), abbreviated T-1. Wernicke’s *zone* includes Wernicke’s area, the supramarginal gyrus (Brodmann area 40), and the angular gyrus (Brodmann area 39). The supramarginal and angular gyri are referred to as the inferior parietal lobule, which lies behind the postcentral gyrus, the site of sensory representation. The Rolandic, or central, fissure separates the frontal from the parietal lobe, and the Sylvian, or horizontal, fissure separates the frontal and parietal lobes from the temporal lobe. The insula is at the depths of the Sylvian fissure, hidden from view. The basal ganglia are subcortical grey matter nuclei between the hemispheric cortex and the lateral ventricle.

### Alexander Luria

Alexander Romanovich Luria was born in 1902 (d.1977) into a well to do family in Kazan, a city in the province of Tatarstan, some 500 miles east of Moscow (Luria, 1974; Homskaya, 2001). His father was a physician and professor of Medicine at the Imperial Kazan University, a well-regarded university where Lenin studied.^2^ The Luria family survived the Russian Revolution, and Alexander’s father was named vice-director of the Kazan Central Institute for Advanced Medical Studies. Alexander completed his secondary schooling in 1918 and matriculated at Kazan University, where he studied philosophy in a futile attempt to gain insight into human psychology, graduating in 1921. He was a laboratory assistant at the Kazan Institute for the Scientific Organization of Labor from 1921, until he was admitted to medical school in 1922. He flirted with psychoanalysis as a medical student, but soon lost faith in its introspective approach. Luria abandoned psychoanalysis for a more objective way of understanding human behavior, based on Pavlov’s application of the conditioned reflex, to study of the central nervous system and psychology. As he wrote, “Pavlovian neurophysiology provided a materialistic understanding to [the] study of the mind” (Luria, 1979), which Luria wished to use to “reconstruct Russian psychology in order to bring it into accord with the goals of the Revolution” (Luria, 1979). In accord with these principles, Luria founded a journal entitled *Problems of the Psychopathophysiology of Labor and Reflexology.*^3^ Luria abandoned his medical studies in 1923 for the position of Head of the Laboratory at the Institute of Experimental Psychology, Moscow University, where he studied the roles of attention, perception, memory, and speech in problem solving. Accordingly, his earliest studies consisted of measuring the reaction time of foundry workers as they responded to verbal instructions, and the influence of emotions on verbal output. His study of language extended from “the relationship of speech and thought, from naming objects to the expression of ideas” (Luria, 1979).

Luria’s dedication to Marxist principles applied to human neurobiology led to his appointment as Director of Psychology at the Krupskaya^4^ Institute of Communist Education, from 1925 to 1934, where he studied the effects of fatigue on the motor reaction-time of workers, but his major interest was in the acquisition of language. There Luria attempted to separate biological and social influences in the development of speech in illiterate and literate children and in twins, and found that the function of language depends on a child’s cultural experience and differing levels of formal education. These studies drew the rebuke of the Institute of Experimental Psychology at its 1931 meeting where Luria’s work was denounced as “apolitically culturist” and “reactionist and inimical to the doctrine of Marxism” (Joravsky, 1990; Homskaya, 2001, 30). Luria would face renewed criticism of lack of Marxist rigor during the Pavlov session of 1950, discussed below.

Luria eventually realized that he needed a greater knowledge of neurology to make any further progress in his research, and he returned to medical school to complete his medical studies. He obtained his M.D. in 1936, a year that stands at the crossroads of his career. In that year he created the Laboratory of Neuropsychology at the Institute of Neurosurgery (the Burdenko Neurosurgical Institute),^5^ where, with the Soviet Union’s declaration of war on Germany in June 1941, his activities were concentrated on the study of post-traumatic aphasia and the rehabilitation of aphasic head-injured patients. His method of study was based on the disruption of what Pavlov referred to as “the cortical sections of the analyzers of the external world” by traumatic lesions (Luria, 1959c, p. 461).

### The acquisition of speech—analyzers and the second signal system

Luria’s physiology and psychology of speech is based on what Pavlov referred to as the *analyzer*, a structure that is central to the creation of conditioned reflexes. Analyzers are aggregations of specialized cortical cells that analyze, synthesise, and coordinate sensory input. They enhance an organism’s responses to certain external stimuli and inhibit its responses to others. In this way, facilitated neuronal pathways are established, and conditioned reflexes are created. As Pavlov wrote, “The nervous system possesses, on the one hand, a definitive analysing mechanism by means of which it selects out of the whole complexity of the environment those units which are of significance, and, on the other hand, a synthesising mechanism by means of which individual units can be integrated in an excitatory complex” (Pavlov, 1927, 110). Pavlov considered excitation and inhibition as equally important in establishing a conditioned reflex (Babkin, 1948). According to Boris Babkin^6^ (1877–1950), his collaborator and biographer, Pavlov “compared the nervous system with the ancient Greek god Janus who had two faces looking in opposite directions” (Babkin, 1949, 313). For Pavlov, as well as for Luria, analyzers were central to the development of speech as an intermediary between humans and their environment: analyzers received auditory and visual stimuli, analyzed and synthesized them, and formulated appropriate verbal or written responses. Pavlov and Luria referred to the output mediated by an analyzer as a signal. The integration of the output of many analyzers – many signals – was required for receptive and expressive speech. It is in this sense that Pavlov referred to speech as a signal of signals, or a second signals system (Windholz, 1990; Chown, 2008).

If our sensations and ideas that pertain to the external world are for us the first signals of reality, namely concrete signals, then speech, especially and primarily kinesthetic stimuli that go to the cortex from the speech organs, are the second signals, namely the signals of signals (Pavlov, 1932, 232)

It is through the second signals system that speech evolved in humans:

The developing animal world on reaching the phase of man acquired an exceptional supplement to the mechanisms of nervous activity. To an animal, reality is signalled almost exclusively merely by the stimulations … conveyed directly to the special cells of the visual, auditory, and other receptors of the organism. This is what we likewise possess in the form of impressions, sensations, and conceptions of the environment, both of the general natural environment and of our social environment, with the exception of words — visible and audible. This first system of signalling reality is the same in our case as in the case of animals. But words have built up a *second system of signalling reality* [emphasis added], which is only peculiar to us, being a signal of the primary signals. The numerous stimulations by word have, on the one hand, removed us from reality, a fact we should constantly remember so as not to misinterpret our attitude towards reality. On the other hand, it was nothing other than words which has made us human (Pavlov, 1941, 179)

Luria also addressed the appearance of speech in human evolution, which he explained, teleologically, in accordance with Marxist doctrine (Chown, 2008). Speech, Luria argued, appeared and evolved from the collective need of early humans to communicate beyond vocalization and gesturing to meet the demands of complex social interactions and the division of labor. As he wrote, “inter-personal division of labor made language necessary… Words became separated from work activities and from signaling gestures; [they] began to abstract, and at the same time to generate, various characteristics of objects… Words gradually became [an] objective system of codes” (Luria, 1970, 20). But for Luria the implications went further. Through this system of codes, thought emerged: “When the designation or sign becomes fixed and becomes a substitute for a concrete phenomenon, when the human being acquires the ability to use these signs instead of using the concrete stimuli, then we are talking about … the physiological basis for thought” (Orbeli, 1964).^7^

### Lurias’s cortical language zones

The analyzers subserving speech settled in cortical *zones*, in Luria’s terminology, in the posterior aspect of the left temporal lobe, the superior and inferior left parietal lobules, and their junction with the occipital lobe, where they recognised phonemes, distinguished them from other ambient sounds, integrated them into words, and formulated verbal responses that were expressed through the action of the muscles of articulation. Despite this broad localization, Luria cautioned that “The speech organization of mental processes must be considered the activity of the brain as a whole, that is the combined work of the whole complex of analyzers” (Luria, 1966, 86). Focal cortical damage might disrupt the function of a single complex of analyzers while others remained intact, but the chain was frayed and broken, and words were perceived as nonsense sounds and graphemes as meaningless scribbles.

Luria recognized three cortical zones involved with speech based on the frequency and severity of the aphasia produced by the injury. These were the *polar* zone, consisting of the anterior-most aspect of the frontal lobe and of the occipital lobe; the *primary* language zone, composed of the inferior aspects of the pre-and postcentral gyri and of the superior aspect of the temporal lobe; and the *marginal* zone, composed of the prefrontal region, the inferior parietal lobule, and the junction of the temporal and parietal lobes with the occipital lobe. Lesions of the primary zone resulted in grossly apparent and complex aphasic syndromes, while lesions of the marginal zone resulted in subtle and limited impairments of speech. Lesions of the polar zone rarely resulted in persistent speech deficits. Distinct aphasic syndromes arose from lesions in specific language zones. These included temporal aphasias, parietal aphasias, and frontal and prefrontal aphasias.

### Traumatic aphasias

Luria began to study aphasic patients at the Burdenko Institute in 1936, and he continued this with renewed energy upon the entry of the Soviet Union in World War Two in June 1941. He recognized that previous studies of aphasia had been conducted in a few elderly individuals with co-morbidities such as arterial hypertension, diabetes mellitus, and other chronic diseases, who had sustained widespread cerebral damage from cerebrovascular or inflammatory lesions. This would not be the case, he reasoned, in healthy young individuals who had sustained focal penetrating craniocerebral injuries. The large number of such cases in wartime, if properly studied, could provide reliable insight into the cortical areas whose damage produced aphasic syndromes, and into the “phonetic, lexical, and logical-grammatical code of language” (Luria, 1959b, 461). Luria gathered a team of well-trained neurologists and psychologists who first assessed patients in field hospitals and followed their evolution until they had achieved maximal recovery and had a fixed aphasic syndrome. From the field, patients were transferred to the Clinic for Nervous Disorders of the Union Institute for Experimental Medicine in Moscow where they stayed until medically stable, and from there to the Neurosurgical Rehabilitation Hospital in Chelyabinsk, in the southern Urals, for rehabilitation (Tables 1, 2)

**Table 1:** Evolution of traumatic aphasia from onset to chronicity.

Zone	Acute deficit	Fixed deficit
Primary	97%	85%
Marginal	80%	47%
Polar	15%	4%

Percentage of patients having sustained an injury to a language zone who had an acute speech deficit and the percentage of those in whom the deficit persisted as a fixed aphasic syndrome. From Luria (1970).

**Table 2:** Luria’s classification of aphasia.

Type of aphasia	Characteristics	Location*	Brodmann areas
**Primary speech zone**			
Temporal acoustic aphasia	Wernicke’s aphasia	Posterior T-1	22
Afferent apraxic motor aphasia	Apraxia	inferior pre-and postcentral gyri	4, 1–3
**Marginal speech zone**			
Efferent kinetic motor aphasia	Broca’s aphasia	Posterior F-3	44
Frontal dynamic aphasia	Abulia	Posterior F-3, orbito-frontal gyrus	45, 47
Superior premotor aphasia	Halting speech	Posterior F-1, F-2	6, 8
Acoustic-mnesic aphasia	Perseveration	Posterior T-2, T-3	21, 37
Semantic aphasia	Anomia, alexia, agraphia, acalculia	Inferior parietal lobule, posterior T-3, temporo-parieto-occipital junction	40, 39, 37

*F- 1, 2, 3 are the first (superior), second (middle), and third (inferior) frontal convolutions, respectively. T-1, 2, 3 are the first (superior), second (middle), and third (inferior) temporal convolutions, respectively. From Luria (1970).

### Luria’s classification of aphasic syndromes

Luria classified aphasia arising from damage to the analyzers of the primary speech zone and from the marginal speech zone. Damage to the primary speech zone was further divided into temporal, or acoustic, aphasia, and afferent or apraxic (motor) aphasia. Damage to the marginal speech zone resulted in a number of speech impairments including premotor, frontal, acoustic-amnesic and semantic aphasias. Each form of speech impairment was localized to a distinct cortical area.

#### Aphasias arising from damage to the primary speech zone

Luria recognized two forms of aphasia arising from the primary speech zone: temporal (acoustic) aphasia, and afferent, or apraxic (motor) aphasia.

##### Temporal (acoustic) aphasia

Acoustic aphasia, to which Luria also referred to as sensory aphasia, occurs with damage to the acoustic analyzers within the posterior aspect of T-1, which includes the *planum temporale*.^8^ Phonemes are no longer discerned from ambient sounds and the meaning of words can no longer be deciphered. As Luria wrote, “Complex sound combinations [phonemes] will be recognized as inarticulate noises and closely similar phonemes will be confused” (Luria, 1966, 73). Acoustic aphasia is synonymous with Wernicke’s aphasia, but Luria does not ascribe this type of aphasia to a deficit of hearing words, as Wernicke suggested, but to a lack of comprehension of their meaning. The patients can speak, but their sentences are confused, jumbled, and meaningless. Incorrect words and paraphasia are common. There are no somatosensory or motor deficits.

##### Afferent (apraxic) motor aphasia

Apraxic aphasia is the only motor aphasia that Luria considered to originate in the primary speech zone. He characterized it as an apraxia resulting from damage to interconnected speech analyzers within the pre-and post central gyri, which normally form a sensorimotor reflex “ring,” or feedback loop.^9^ Luria distinguished apraxic speech, in which articulation is preserved but disorganized, from dysarthric speech, in which words are mispronounced and slurred. Their common feature is a deficit in the motor output of speech.

#### Aphasias arising from the marginal speech zone

Luria considered that the marginal aphasic zone encompassed: 1- the premotor area, 2- the inferior-posterior aspect of the second temporal convolution and the posterior aspect of the third temporal convolution, and 3- the parietal lobe in front of the precentral gyrus.

##### Premotor aphasias

The premotor region is defined as that part of the cerebral hemisphere that resides between the precentral gyrus and the frontal pole. The lower premotor region –Boca’s area – is opposite the motor representation of the face, lips, and the muscles articulation. The mid-portion of the prefrontal region is in front of the motor hand and arm representation. It is within the pre-motor region that motor responses related to spoken and written words are organized. Damage to the premotor analyzers results in: i- *efferent motor aphasia* (Broca’s aphasia), ii- *frontal dynamic aphasia*, and iii- *disorders of automatic speech*. The severity of each of these syndromes is a function of the distance of the causative lesion to the precentral gyrus.

###### Efferent (kinetic) motor aphasia — Broca’s aphasia

*There is no basis whatever for the idea that … Broca’s area … is anything other than a highly specialized part of the premotor area* –Luria.^10^

This syndrome arises from damage to Broca’s area, which Luria considered to be part of the marginal speech zone. This area is immediately in front of the motor representation of the face, lips, and the muscles of articulation, from which it receives afferent fibers. It also receives afferent fibers from the primary temporal speech area in what constitutes a functional receptive-expressive system. In Luria’s model, Broca’s area is the final common pathway for the generation of speech. Disruption of Broca’s area renders the speaking of words impossible although the muscles of articulation function properly. Patients can pronounce individual sounds –such as *tan* – but they are unable to organize sound into a chain to complete a word. This type of aphasia is almost always accompanied by damage to the pre-and post central gyri causing sensorimotor deficits.

###### Frontal (dynamic) aphasia

This form of aphasia arises from damage in the marginal zone in front of Broca’s area. It results in the inability to initiate spontaneous speech, although the ability to correctly repeat words and sentences is preserved. Luria characterizes this type of aphasia as a lack of “verbal initiative” (Luria, 1970, 202).^11^ It corresponds to the *abulia* commonly seen with damage to the orbito-frontal area from the rupture of an anterior communicating artery aneurysm, and, indeed, this had occurred in one of Luria’s index cases (Luria and Tsvetkova, 1968).

###### Superior premotor aphasia

Patients with this type of aphasia can fully articulate their thoughts, but they do so haltingly, hesitantly, because of damage to F-1 and F-2 in front of the precentral gyrus.

##### Acoustic-mnesic aphasia

This form of aphasia arises from damage to the inferior-posterior aspect of T-2 and to the posterior aspect of T-3. It is characterized by the inability to retain the meaning of words, and the inability to speak a short series of words. This leads to repetition, perseveration, and paraphasia. Luria considered this type of aphasia as a deficit of short-term memory.

##### Semantic aphasia

Semantic aphasia^12^ results from damage to the posterior aspect of T-1 and the inferior parietal lobule, which contains many analyzers related to tactile, auditory, and visual functions. As in primary temporal (acoustic) aphasia, the patient speaks, but does so incoherently. Anomia, alexia and agraphia arise from the inability to integrate phonemes or graphemes into sequences of letters comprising a word or a sentence, and acalculia arises from the inability to combine individual digits into numbers. The presence and severity of anomia, alexia, agraphia, and acalculia depends on the proximity of the lesions to the parietal-occipital and the temporal–parietal-occipital junctions (Luria, 1959a,b,c, 1966, 1970; Dragoy et al., 2017).

### Paradoxical aphasia and hemispheric dominance

Since the seat of articulate language was found to be in the left hemisphere of right-handers, it was commonly assumed that the converse was also true, that speech was represented in the right hemisphere of left-handed individuals.^13^ That this relationship was not as straightforward as was commonly thought was raised by Luria when he encountered right-handed patients who had sustained an injury to the left hemisphere but who were not aphasic, and, rarely, right-handed patients who were aphasic after an injury to the right hemisphere. Looking deeper into these occurrences, Luria compared the incidence of aphasia in a series of patients who were right-handed and had a family history of left-handedness, who were ambidextrous, or who were purely left-handed, to a cohort of purely right-handed individuals (Luria, 1970, 69–70). He observed that pure right-handers who had sustained an injury to the left hemisphere rarely escaped aphasia, while right-handers with a family history of left-handedness or ambidextrous individuals sustaining similar injuries in the left-hemisphere were usually not aphasic or only mildly so. This led Luria to formulate a novel theory that held that hemispheric dominance for speech is a continuum:

a whole series of intermediate stages ranging from absolute dominance by the left hemisphere, through equivalence of the two hemispheres, to dominance by the right hemisphere may be expected to occur. It also means that parts of the right hemisphere which are symmetrical with the speech areas of the left hemisphere may be in large measure capable of assuming the functions of the left hemisphere if it is damaged… The ease with which it occurs appears to depend upon the extent to which various individuals possess the genotypic potential for the right hemisphere to fulfill the complex functions which are usually performed by the left (Luria, 1970, 62–63).

Luria was only partially correct. The relationship of handedness and cerebral dominance for speech was worked out at the Montreal Neurological Institute by Brenda Milner and Theodore Rasmussen using the intracarotid injection of sodium amytal (Wada and Rasmussen, 1960). They found that only 15% of left-handed individuals are right hemisphere dominant for speech (Rasmussen and Milner, 1975) and that transference of speech to the right hemisphere only occurs in individuals who have sustained severe damage to the left hemisphere in childhood (Rasmussen and Milner, 1977). The genetics of handedness remain to be elucidated (Annett, 2002).

Luria’s classification of traumatic aphasias shared some syndromes with previous classification schemes (Geschwind, 1972; Valdois et al., 1989). The noted aphasiologists Norman Geschwind (1926–1984) and Roch Lecours (1936–2005) agreed with Luria that his temporal (acoustic) aphasia and efferent (motor) aphasia correspond to Wernicke’s and Broca’s aphasias respectively. Geschwind also agreed with Luria that his afferent (apraxic) motor aphasia corresponds to Kurt Goldstein’s (1878–1965) transcortical aphasia (Goldstein, 1927), but Lecours did not. As for semantic aphasia, Lecours thought that Luria’s delineation excluded some of the elements that Henri Head (1861–1940) had included in his description of the syndrome (Head, 1926). Geschwind did not address Luria’s other syndromes, but Lecours recognized them as distinct entities.

### The Pavlov session

Luria continued his work at the Burdenko Institute after the war, focusing on the rehabilitation of aphasic patients, but his work, and his life, were put in jeopardy in the summer of 1950. Concerns had been raised among the upper echelons of the Soviet intelligentsia, driven by Stalin himself, that Soviet science had strayed from Pavlovian principles and had embraced Western scientific concepts, in what was referred to derogatorily as *Cosmopolitanism.* The first blow to bourgeois science and cosmopolitanism came in 1948 when Trofim Lysenko (1898-1976) purged the governance of the Academy of Agriculture and forced the application of Lamarckian theory to Soviet agriculture (Pollock, 2006). The next blows came concurrently in the fields of linguistics and of physiology, which included Luria’s work on aphasia (Stalin, 1950).^14^ A combined meeting of the Academy of Sciences and the Academy of Medical Sciences of the USSR was held in Moscow from June 28 to July 4, 1950, in what is known to historians as the *Pavlov Session*, under the personal direction of Stalin and his son-in-law Yuri Andreievich Zhdanov (1919–2006), head of the Central Committee of the Communist Party of the Soviet Union’s Science Section (Gordon, 1951; Brushlinsky, 1997; Windholz, 1997; Homskaya, 2001; Pollock, 2006; García-Molina and Peña-Casanova, 2022). Luria and others, most notably Leon Abgarovich Orbeli (1882–1958), director of the Institute of Physiology and Pathology of the Higher Nervous System, were accused of having deviated from Pavlovian principles and of lacking a Soviet approach to the study of psychology. More specifically, they were accused of having made “little effort … to develop Pavlov’s concept of the ‘second signals system,’” especially as it applied to speech (Joravsky, 1990; Homskaya, 2001; Pollock, 2006, 151,153).^15^ Despite his membership in the Communist Party, his self-denunciation, and his refutation of Charles Sherrington’s (1857–1952) *Integrative action of the nervous system* (1906), Luria was dismissed from his position at the Burdenko Institute and reassigned to the Institute of Defectology^16^ of the Pedagogical Sciences (Knox, 1989; Joravsky, 1990). Despite this change of fortune, Luria continued to study the role of speech in the intellectual development of children, as he had done at the beginning of his career, and introduced novel physiological technologies, such as the EEG, in his research, most notably in twins and veterans who had sustained a head injury (Luria, 1958, 1959a,b, 1961, 1963). Luria was eventually rehabilitated, and his work became widely known in the West, where it was received to wide acclaim (Luria, 1966, 1968, 1970, 1972, 1973, 1974).^17^

The Pavlov session was as devastating to Soviet physiology and psychology as Lysenkoism was to Soviet genetics and agriculture. The cream of Soviet experimentalists in physiology, psychology, and medicine were dismissed from their positions, and replaced by their accusers who foreswore western science in favor of Pavlovian neurophysiology, which by then had long past its usefulness in experimental neurobiology. As a prominent Russian historian put it:

The 1950 session not only prevented the development of physiology and medicine, but was a great blow to the moral foundations of science. It destroyed the futures of many scientists, and distorted the psychology of the youth, by encouraging their servility and immorality. It distorted the spirit of the physiology of higher nervous activity and disseminated dogma, conformism, and that monolithic spirit that is so inappropriate in science (Grigorian, 1989).

A Scientific Advisory Bureau was formed in 1951 to review research proposals to assure that researchers did not stray from the path charted in the Pavlov session. A separate meeting of representatives of Soviet psychology met in 1952 and called for the reorientation of the discipline along Stalinist lines. One of the research projects went so far as to propose to study the differences between conditioned reflexes in Soviet citizens and those of the bourgeoisie (Windholz, 1997). Soviet science remained bound within the straitjacket with which it was fitted in the Pavlov session until 1962, when it was freed at the Soviet Union Conference on Philosophical Issues of Physiology of Higher Nervous Activity and Psychology (Brushlinsky, 1997).

Wilder Penfield (1891–1976) was aware of the Pavlov session, but seemed to minimise its excesses, as he wrote following a two-week trip to the Soviet Union, in January 1955, when he visited the most prominent biological research institutes in Moscow and Saint Petersburg:

Scientists outside the Soviet Union have wondered about its outcome. Many were under the impression that a trial had been staged rather than a free discussion. This was apparently a misconception, for no penalties were imposed. Some physiologists were accused of a defect in their loyalty to “Pavlov’s materialistic teachings,” or of being influenced by the “dualist and animist” point of view or of worshipping “foreign science and cosmopolitanism.” I can testify to the fact that all of these physiologists, involved in this discussion five years ago, are continuing to carry on constructive scientific work, without interruption and with no lack of facilities (Penfield, 1955, 892).

Penfield’s stunningly naive view of biological research under the Soviet regime undoubtedly arose from his meetings with heads of institutes who had been the denunciators of cosmopolitanism,^18^ and others who had been forced to recant their “dualism and vitalism.”^19^ In any event, all the institutes that Penfield visited were engaged in research on conditioned reflexes, which could only have reinforced his reliance on Pavlovian conditioning to explain neurophysiological phenomena.

Luria continued to work on his classification of aphasic syndromes until his death (Luria and Hutton, 1977), and it continues to be of interest, especially in Russia (Akhutina, 2016; Ardila et al., 2020; Markashova et al., 2021).

### Wilder Penfield: beyond lesion analysis

*This institute is housed in a curiously curved building on the Neva River. From the front door a grand staircase of white marble leads upward to the second floor. As we climbed, I could hear the faint echo of the barking dogs and I thought of how often Pavlov had himself climbed those stairs and savoured familiar smells and sights and sounds. A superstitious dualist, if one had been present, might have fancied that the spirit of Pavlov was walking with us through the laboratory* — Penfield, 1955^20^

Wilder Penfield is widely acknowledged for his elucidation of the structure–function relationships of the brain, and the surgical treatment of focal epilepsy (Leblanc, 2019a). He became acquainted with Pavlov’s work on the conditioning of functionally specific areas of the cerebral cortex through Gleb Vasilievitch von Anrep’s^21^ (1891–1955) translations of Pavlov’s lectures on conditioned reflexes, given at the Military Medical Academy in Petrograd (Saint Petersburg) in 1924 (Pavlov, 1927). His introduction to the Pavlovian physiology of speech, though, was more personal, through an exchange with Boris Babkin in 1936, who informed him that “if a conditioned stimulus is regarded as a signal, there also may be formed ‘signals of signals,’ e. g., words” (Penfield, 1938, 438; Leblanc, 2019b). Penfield never forgot Babkin’s short introduction to the Pavlovian foundations of speech, which he later echoed when he stated, “Speech might be looked upon as a special conditioned reflex. In a certain sense, it is a collection of conditioned reflexes that enables a man to write and to speak and to use that special skill that makes possible the interpretation and the understanding of the spoken and written word” (Penfield, 1969).

Luria’s work on aphasia was largely unknown in the West until the publication of his paper *Brain disorders and language analysis*, in 1958 (Luria, 1958). The following year Penfield and Lamar Roberts (1919–1978) published their epoch-marking book *Speech and Brain Mechanisms* (Penfield and Roberts, 1959). Although it is unlikely that Penfield was aware of Luria’s *Traumatic Aphasias* published in Russian in 1947, Luria was certainly aware of *Speech and Brain Mechanisms* since he cited Penfield’s and Rasmussen’s *Cerebral Cortex of Man* (1950) in the English edition of *Traumatic Aphasias* published in 1970. Inexplicably, he does not refer to it.

Penfield became interested in speech in 1935 when he discovered that electrocortical stimulation of either inferior precentral gyrus – corresponding to the face area – produced vocalization, which he defined as “a clear, sustained vowel cry” (Penfield, 1938; Penfield and Rasmussen, 1949; Leblanc, 2019a). The study of the localization of speech at the Montreal Neurological Institute, however, began in earnest with Penfield’s research fellows Preston Robb (1914–2004) and Lamar Roberts.

#### Preston Robb

Preston Robb graduated from McGill Medical School in 1939. He trained in neurology at the Montreal Neurological Institute (MNI) where he was appointed neurologist-in-chief in 1968. As was required at the time, those wishing to train in neurology or neurosurgery at the MNI had to spend at least 1 year in research before starting clinical training. Robb’s research came at a turning point in Penfield’s interest in language. Prior to 1946, Penfield attempted to delineate the cortical representation of speech by injecting the local anaesthetic nupercaine into the cortex of classically defined speech areas at operation. This failed to interfere with speech, and worse, it created small cysts within the cortex. Penfield’s attempts to generate an aphasic response by applying pressure to the cortex with the ball of his thumb also proved ineffective. By the time Robb arrived at the Institute Penfield had begun to stimulate the cortex with an electrical current while the patients named small objects held in the hand or depicted on index cards. If the patients were unable to name the object or its image, they were asked to describe its use verbally or by pantomime. On some occasions, the patients were also asked to read and to count (Leblanc, 2019a).

Robb undertook the study of the effects of cortical injury on speech for his master’s thesis entitled *The effect of cortical excision on speech in patients with previous cerebral injury* (Robb, 1946). Robb’s patients, however, were unlike Luria’s as none had sustained a gunshot wound to the head. Rather, their cortical injuries were incurred in civilian life, and many had sustained focal cortical injuries from a traumatic passage through the birth canal. All the injuries were within or near classically defined language areas. Robb also included the first patients in whom Penfield had delineated the speech areas through electrocortical mapping. Robb published his observations in his thesis, and in a presentation to the *Association for Research in Nervous and Mental Disease* in 1947 (Robb, 1946, 1948). Robb’s presentation is noteworthy because it includes the first composite brain map illustrating the responses obtained from stimulation of Broca’s area, Wernicke’s area, and the inferior parietal lobule (the role of the supplementary motor area in speech had not been discovered yet). His research year having come to an end, Robb continued his clinical training in neurology and epileptology, and Lamar Roberts continued where Robb had left off.

#### Lamar Roberts

Lamar Roberts graduated in medicine from Duke University in 1944. He began his training in neurosurgery at the MNI in 1945, and stayed there as surgical resident, research fellow, and associate neurosurgeon until 1959. Roberts, in Robb’s wake, studied the effects of resection and electrocortical stimulation of putative speech areas for his master’s thesis and doctoral dissertation. His work formed the backbone of *Speech and Brain Mechanisms* (Roberts, 1949, 1952; Penfield and Roberts, 1959).^22^ But Roberts expanded his reflections on speech with cogent discussions on the relationship of handedness and speech and on cerebral plasticity (Roberts, 1955).

**Figure 2:**
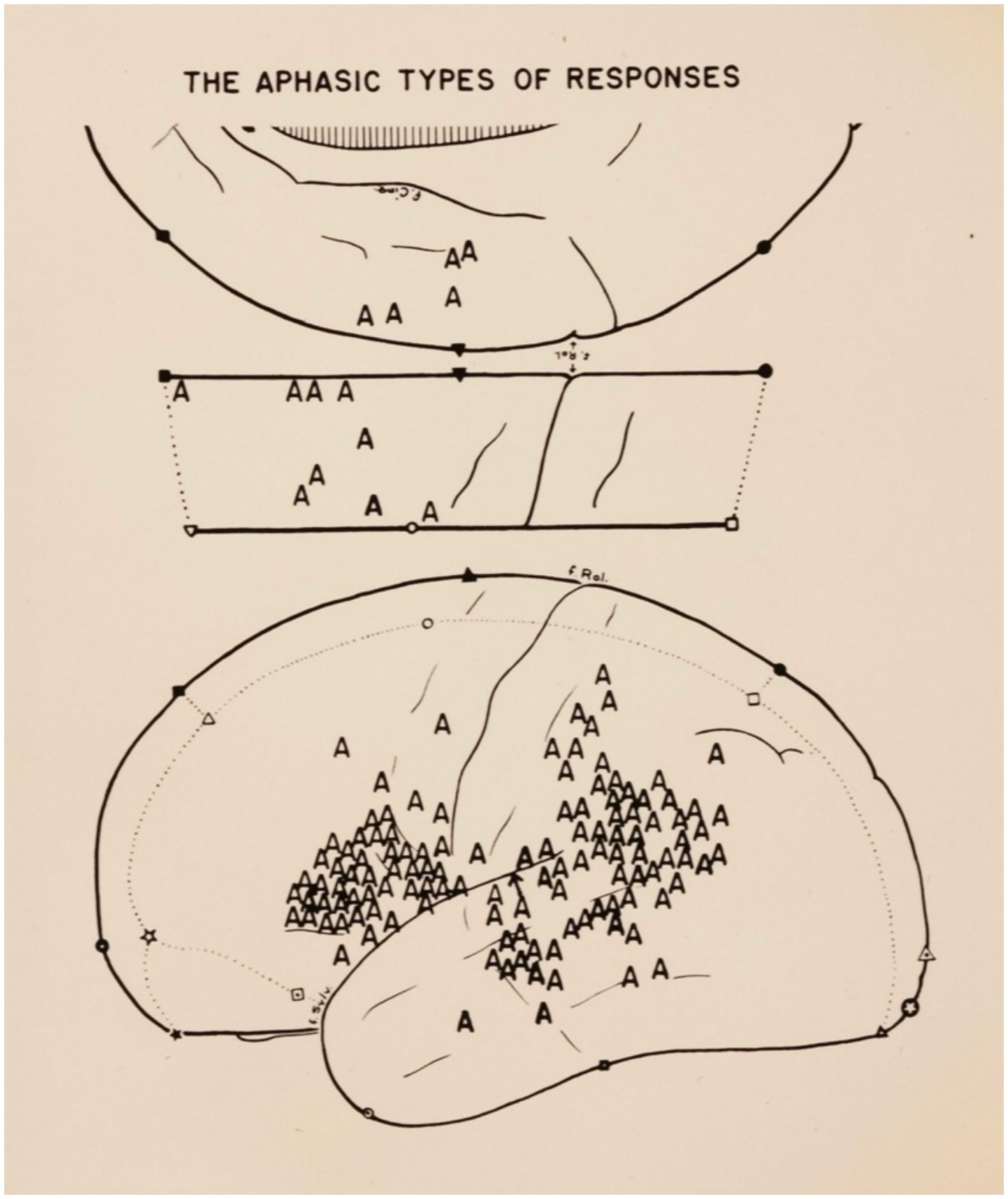
Roberts cumulative brain map showing areas of aphasic responses (A). From Roberts (1952). © Used with permission of the Montreal Neurological Institute Archives.

Roberts’ studies included patients who were instructed to speak, read, write, and count while the exposed cortex was stimulated with a blunt electrode that delivered a mild electrical current. He then classified their responses as aphasic or non-aphasic. Non-aphasic responses were obtained from Broca’s area and the inferior premotor region of either hemisphere. They consisted of interference with the motor mechanisms of speech characteristic of anarthria and of dysarthria, including speech arrest, hesitation, slurring, distortion, and repetition. Aphasic responses were obtained by interference with the cognitive aspects of speech – Roberts refers to this as “the language process of speech” – which consisted of the inability to name objects, misnaming objects with or without perseveration, and errors in counting (Figure 2). They were elicited by stimulation of the left hemisphere within Broca’s area, Wernicke’s area, the supramarginal and angular gyri, and the SMA^23^ (Table 3).

**Table 3:** Penfield and Roberts classification of speech alterations.

**Non aphasic**	
Inability to vocalise or speech arrest	Inferior precentral gyrus, inferior premotor (Broca’s area), supplementary motor area
Hesitation and slurring	Inferior premotor region (Broca’s area)
**Aphasic**	
Distortion of words and repetition of words and syllables	Inferior precentral gyrus and posterior T-1
Inability to name with retained ability to speak	Posterior T1, T-2, inferior parietal lobule
Misnaming with and without perseveration	Posterior T-1 and T-2, inferior parietal lobule, mid-and inferior premotor region
Confusion of numbers while counting	Inferior premotor region, posterior T-1

From Penfield and Roberts (1959).

Like Luria, Penfield and Roberts found it difficult to define the role of Broca’s area as a specific speech center. As Roberts put it in his thesis, “lesions of the region of the precentral face area and of Broca’s area may result in aphasic disorders which are predominantly expressive” (Roberts, 1952, 182). Adding to the confusion, responses characteristic of Broca’s aphasia were not restricted to Broca’s area: “the transmission of impulses from the precentral gyrus to all of the complex musculature necessary for speech is certainly occurring,” Roberts observed, but continued, “there is, however, no localized area for *articulate language* [emphasis added] in Broca’s convolution. Broca’s convolution is only part of the whole” (Roberts, 1952, 183). Thus, contrary to Broca, who localized motor aphasia to Broca’s area, Roberts observed that motor aphasia also occurs with lesions in Wernicke’s area or within the inferior parietal lobule. This does not mean that all aphasias were the same, as Penfield and Roberts observed: “there are definite differences in the types of aphasia produced by lesions in different portions of the speech cortex. In some cases, there is more involvement of the sensory side of speech, and in others, more of the motor elements. Thus, there is what clinicians have called *motor aphasia*, in which speaking is severely involved while understanding of speech is relatively and comparatively intact. There is also *sensory aphasia* in which the reverse is true” (Penfield and Roberts, 1959, 247). The predominance of motor or sensory aphasias was related to the proximity of the lesion to the anterior or posterior speech zone, respectively.

### Speech zones

Penfield and Roberts recognized three speech zones in the left hemisphere: the anterior speech zone, corresponding to Broca’s area, the posterior speech zone, corresponding to the posterior aspects T-1 and T-2 (Wernicke’s area), and the superior speech zone corresponding to the SMA. The three speech zones were integrated into a functional whole as their cortices were joined through cortico-cortical and thalamo-cortical fiber tracts. Stimulation or damage of any part of the system disrupted the function of the system as a whole. The thalamus, in Penfield’s model, was central to the integration of ideational and motor speech through fiber tracts coursing between the premotor area, the temporal lobe, the inferior parietal lobule, and the SMA and reciprocal thalamic nuclei.

Penfield and Roberts ranked the speech zones according to the long-term effects on speech of damage to each specific zone. Complete but temporary mutism occurred from resection within the SMA, from which patients completely recovered within one week to ten days after surgery. Penfield rarely resected Broca’s area but often resected cortex in its proximity. This resulted in aphasia that also improved, but over a longer period of time. Nonetheless, Penfield and Roberts advised against resection of Broca’s area. Resection within the posterior language zone produced permanent aphasia. Penfield and Roberts concluded from these observations that, “the most important area for speech is the posterior temporo-parietal region, including the posterior parts of the first, second, and third temporal convolutions, … the supramarginal gyrus, and the angular gyrus. The next important area for speech is that of Broca… The supplementary motor area on the medial, and a little on the superior aspect of the hemisphere in front of the precentral foot area is dispensable” (Penfield and Roberts, 1959, 188).

### The acquisition of speech

In Penfield’s view, vocalization played a critical role in the evolution of speech. Vocalization for Penfield is more than an animal’s call, and the interaction of the precentral gyrus from which vocalization arises, and the newly evolved prefrontal region, is unique to humans. “Without the help of man’s vocalization projection to the newer cortex,” he wrote, “it is quite possible that vocalization could not be used in the complicated patterns which he employs for human speech” (Penfield and Roberts, 1959, 238). But vocalization alone was insufficient for the development of human speech. This required the disproportionally large increase of the parieto-occipital cortex, which paralleled the increase in the size of the frontal lobes, as we evolved from sub-human primate to *homo sapiens*. Ideational speech would then have settled in the posterior part of the left hemisphere, interposed between the sensorimotor, auditory, and visual cortices, and Broca’s area. Penfield was more specific for the timeline of the appearance of writing, which he dated to the development of agriculture “when man learned to cultivate grain in the valleys of the Nile and the Euphrates.” This required the interplay of symbols related to ideational speech, motor function, and arithmetic. As Penfield saw it, “a tablet of soft clay was held in the palm of one hand while the scribe printed the cuneiform letters onto its surface, using a sharp stylus held in the other hand” (Penfield and Roberts, 1959, 239). Penfield, as did Luria, proposed a teleological view of the origins of speech and writing, in that a societal need created the organs of speech, when it is more likely that the appearance of speech during the late Pleistocene created communal societies (Jaynes, 1976; Lieberman, 2000).

Physiologically, Penfield believed that humans at birth possess two functionally different cortices, the committed cortex and the uncommitted cortex. The committed cortex is composed of the pre-and-postcentral gyri, the occipital lobes, and Heschel’s gyri, which are innately committed to sensation, motility, vision, and audition. The other type of cortex is uncommitted at birth, a white paper upon which nothing is as yet written (Leblanc, 2019c). It consists of the premotor regions, the parietal lobes behind the postcentral gyri, and the temporal cortex bilaterally. With maturation and conditioning, these regions develop memory, perception, speech, and the will to act towards a goal. In this way, the initially uncommitted cortex matures into what Penfield named the *interpretive cortex* (Penfield, 1959, 1965a,b). The interpretive cortex of the left hemisphere subserves language. The remaining interpretive cortex of both hemispheres subserves memory and perception, with perception defined as the interpretation of the present in light of past experience. Speech and perception were the result of the conditioning of the uncommitted cortex, as Penfield wrote in *Science* in 1959 and *Brain* in 1965: there is “a large area of cortex covering a given, large part of each of the two temporal lobes that is uncommitted at birth. This uncommitted cortex will in time be used for language and for perception. For language, it will make possible the remembrance and use of words. For perception, it will play a part in the recall of the past and the interpretation of present experience” (Penfield, 1965b, 789).

Penfield thought that these functions — memory, speech, perception — came about through Pavlovian conditioning. Like Pavlov, Penfield considered that humans have both inborn and acquired reflexes. The former include, for example, stretch reflexes and the chain of reflexes responsible for maintaining the upright posture. More evolved aspects of human behavior, such as speech, depend on the establishment of acquired reflexes within distinct areas or zones of the cerebral cortex. For Penfield, speech, “might be looked upon as a special conditioned reflex. In a certain sense, it is a collection of conditioned reflexes that enable a man to write and to speak and to use that special skill that makes possible the interpretation and the understanding of the spoken and the written word” (Penfield, 1969, 149). As for speech, so for perception:

Speech and perception depend upon acquired ideational mechanisms established by the child in this great zone of cortex [the interpretive cortex]. The mechanisms come to serve the adult as an aid to interpretation—interpretation of speech posteriorly on the dominant side, interpretation of experience in the light of the individual’s past in the other portions of both sides … Thus, an area on one side, the speech cortex, is conditioned for speech. The remainder of the initially uncommitted cortex … is programmed to serve the purposes of perception” (Penfield, 1969, 143, 149)

For Penfield, conditioned reflexes, words, memory, and thoughts were inexorably linked. Hearing or reading a word simultaneously evoked the idea of an object, the inner image that the word represents. Similarly, a thought immediately brought forth the corresponding word “by acquired automatic reflex action.” And here, one is allowed to link both Pavlov’s and Penfield’s materialism as he states, “the connection between *speech mechanism* and *concept mechanism* is evidently reflex and automatic” (Penfield and Roberts, 1959, 234). Speech, memory, and thoughts were the material foundation of Penfield’s *physiology of the mind* (Leblanc, 2019a,b,c).

### The physiology of mind

Penfield went further than Luria’s association of word and thought. He proposed a theory uniting brain, consciousness, memory, speech, perception, and willed behavior toward a goal through a bidirectional, functional pathway between the cortex and the thalamus, which he called the *centrencephalic integrating system* (Penfield, 1958; Leblanc, 2019a,c). By consciousness, Penfield did not mean only a state of alertness, rather, it is “an awareness, a thinking, a knowing, a focusing of attention, a planning of action, an interpretation of present experience, a perceiving. … [an] integrated perception of the present” (Penfield and Roberts, 1959, pp. 38–39). Consciousness brought about self-awareness, which Penfield considered to arise with conditioning and maturation of the interpretive cortex of the right parietal lobe, behind the post-central gyrus (Hécaen et al., 1956). Memory also had an anatomical substrate comprised of the lateral neocortex and the mesial archicortex of the temporal lobes. Memory, acquired through conditioned interaction with the external environment, made the acquisition of language possible, and guided willed behavioral responses based on past experiences. These functions of the human brain were fundamental to a person’s interactions in society and were acquired through learning based on conditioning. Pavlov, Penfield noted, had said “learning is a process based on the establishment of conditioned reflexes in the cerebral cortex [of dogs],” which, Penfield added, is “valid for man” (Penfield, 1969, 136). For Penfield, then, what are generally considered as attributes of the mind were localized to specific areas of the brain, and were accounted for by conditioned reflexes, as materialistic a view as Pavlov might have held.

## Discussion

Luria and Penfield reignited interest in the localization of speech and in aphasiology, while also proposing a neural mechanism by which speech is acquired based on Pavlovian conditioning.

Luria added little to what was already known about aphasia from the work of Broca and Wernicke on the localization of expressive and receptive speech deficits, and Dejerine’s on the localization of alexia with or without agraphia (Dejerine, 1891a,b, 1892); Geschwind, 1972 (Figure 1). Luria’s integration of functional speech-competent areas through subcortical connecting fibres was also derivative, as it had been suggested by Theodor Meynert (1833–1892) and Carl Wernicke, and demonstrated by the Dejerines’ discovery of the arcuate and superior longitudinal fasciculi (Dejerine and Dejerine-Klumpke, 1901; Whitaker and Etlinger, 1993). Even Luria’s inclusion of the prefrontal cortex above and anterior to Broca’s area in the expression of speech had been presaged by Sigmund Exner (1849–1926) (1881), and by Jean-Martin Charcot (1825–1893) who had suggested that agraphia was caused by a lesion of the posterior aspect of the second frontal convolution in front of the motor hand area.^24^

Luria relied on a projectile’s point of entry through the skull to localize the site of cortical damage resulting in aphasic syndromes, assuming that the damage was limited to the immediately underlying cortex. This will seem dubious to anyone treating traumatic brain injuries. Luria also realized this methodological problem and tried to circumvent it in two ways: first, he waited until sufficient time had passed for the secondary effects of the initial injury – brain swelling, hemorrhage – to abate and for the deficit to become fixed. However, the difficulty in discrete localization remained because, even if the hematoma had resorbed, so had the associated tissue, leaving a post-traumatic cavity. Similarly, Luria failed to account for possible damage to the brain distant from the point of entry caused by *contrecoups* lesions, or from the intracranial ricochet of low velocity projectiles as they hit the inner table of the skull. Second, Luria claimed that the study of many cases would dampen the “background noise” from secondary brain damage and that only the “peaks” of the initial site of injury would remain for his correlation with aphasic deficits. One suspects that Luria’s cases were still too few to transform a multitude of diffuse and disparate cortical injuries into a standardized lesion from which generalizable conclusions can be made.

This was not the case with Penfield’s electrocortical mapping, as he applied a blunt electrode tip to the cortex. The sites of positive and negative responses were identified by small numbered paper tickets that were applied to the cortex. The cortex was then photographed, and the sites from which interference with speech had occurred were correlated to the Sylvian and Rolandic fissures. The positive responses obtained from all the patients were transposed onto a composite brain map and appeared as a tight scatter plot on which the sites where positive responses were most consistently obtained became apparent (Figure 2).

Luria introduced the vast premotor region beyond Broca’s area to aphasiology, which Penfield and Roberts were unable to confirm. Penfield did, however, discover the speech-competency of the supplementary motor area, the most significant advance in the neurobiology of speech since Dejerine’s localization of alexia and agraphia to the angular gyrus.

Penfield and Roberts recognition that specific aphasic responses are not limited to specific speech-competent regions is an important feature of their work. Aphasic expressive motor responses were generated not only from Broca’s area, but also from stimulation of the inferior precentral gyrus. Similarly, interpretive aphasic responses were generated from stimulation of the posterior temporal lobe and the inferior parietal lobule, as well as from Broca’s area. Penfield and Roberts explained these occurrences by reciprocal cortico-cortical interconnections and interconnections between cortex and the thalamus and upper brainstem, in what Penfield referred to as the centrencephalic integrating system (Penfield, 1958; Leblanc, 2019a).

Commentators of Luria’s and Penfield’s work have failed to appreciate that they went beyond localizing different aspects of speech to different brain regions: they sought to explain how speech arises at the neural level, which they found in Pavlovian conditioning. In doing so, they unified the anatomy and neurology of speech and aphasia with the basic mechanism by which speech arises. In essence, they provided a comprehensive theory of speech that explained how an attribute of the mind can arise from the brain without turning to metaphysics. Penfield went further than Luria in that he explained how other attributes of mind – memory, perception, will – also arise from conditioned reflexes. It is true that Penfield, at the end of his life, and after struggling “to prove that the brain accounts for the mind,” turned to dualism. But this was perhaps more from a sense of despondency than spiritual insight. As Herbert Jasper (1906–1999), Penfield’s friend and collaborator explained: “Penfield finally concluded, in his last publication, *The Mystery of the Mind*, that the brain-mind problem could not be solved since he had spent his entire life trying to do so without success” (Penfield, 1975; Jasper, 1986, unpaginated; Leblanc, 2019c, 426–427).

Contemporary historiography has yet to recognise that Luria and Penfield went beyond the localization of speech and the manifestations of aphasia. They attempted to answer the ultimate question: how do we come to speak?

## Data availability statement

The original contributions presented in the study are included in the article/supplementary material, further inquiries can be directed to the corresponding author.

## Ethics statement

Ethical approval was not required for the study involving humans in accordance with the local legislation and institutional requirements. Written informed consent to participate in this study was not required from the participants or the participants’ legal guardians/next of kin in accordance with the national legislation and the institutional requirements.

## Author contributions

RL: Writing – review & editing, Methodology, Investigation, Writing – original draft, Project administration, Formal analysis, Data curation, Conceptualization.

## Footnotes

^1^Pavlov (1928, 179).

^2^A victim of changing times, the Imperial Kazan University was renamed the V. I. Ulyanov-Lenin Kazan State University in 1925. It is now known as the Kazan Federal University.

^3^i.e., classical conditioning.

^4^The Institute was named in honor of Lenin’s wife Nadezhda Konstantinovna Krupskaya (1869–1939).

^5^Nikolai Nilovich Burdenko (1879–1946) was a neurosurgeon, and Surgeon-in Chief of the Red Army during the Second World War.

^6^Babkin was born in Kursk, Russia, in 1877 (d. 1950). He began work with Pavlov in 1902 and performed the first experiments on cortical conditioned reflexes with him at the Institute of Experimental Medicine in St. Petersburg, Russia. Babkin was appointed professor of physiology at the University of Odessa in 1915, but in 1922, his revolutionary fervor deemed lacking, he was sentenced to a term in prison before being exiled from the Union of Soviet Socialist Republics. Babkin took refuge in Ernest Starling’s laboratory at University College, London, UK, before being appointed Chair of Physiology at Dalhousie University, Halifax, Canada in 1924. He was appointed Professor in the Department of Physiology at McGill University in 1928, and Chair in 1941. Babkin came to the MNI as a Senior Research Fellow in Neurophysiology after his retirement from the professorship at McGill and worked there until his death in 1950. His interests at the Institute were on the role of the cerebral cortex and subcortical centers in the regulation of gastrointestinal function, and on the function of the frontal lobes (Feindel and Leblanc, 2016).

^7^Leon Abgarovich Orbeli (1882–1958) director of the Institute of Physiology and Pathology of the Higher Nervous System (1936–1950), and the Soviet Union’s most prominent physiologist after Pavlov’s death (Windholz, 1990).

^8^The *planum temporale* is larger in the left temporal lobe in most individuals (Geschwind and Levitsky, 1968).

^9^Luria based this interpretation on Penfield and Rasmussen’s findings that motor responses can sometimes be elicited from stimulation of the postcentral (sensory) convolution. (Penfield and Rasmussen, 1950)

^10^Luria (1970, 174).

^11^Geschwind (1972) and Lecours and Lhermitte (1979) consider frontal dynamic aphasia to correspond to Goldstein’s motor transcortical aphasia. Of note, Luria met Goldstein in Berlin, in 1925 (Homskaya, 2001).

^12^Luria credits Henry Head (1861–1940) for defining semantic aphasia (Head, 1926; Luria, 1970, 240). Head characterized semantic aphasia as the inability to “formulate accurately, either to himself or to others, a general conception of what he has been told, has read to himself, or has seen in a picture, although he is able to enumerate most of the details. Such patients understand what is said, can read and can write, but the result tends to be inaccurate and confused. Counting is possible and the relative value of coins may be recognized, but arithmetical operations are affected… and orientation is definitely disturbed” (Head, 1926, 2, xix- xx).

^13^Jackson reported the first case of a left-handed man with a lesion of the right hemisphere and aphasia; however, he probably did not know it was the first case, as he seemed to have accepted the theory that aphasia would accompany disease of the right hemisphere in the left-handed (Penfield and Roberts, 1959).

^14^Stalin’s personal involvement in promoting Pavlovian science as the only science was based on the notion that controlling the stimuli and conditions of Soviet society would lead to the creation of a New Soviet Man (Soboleva, 2017) dedicated to collectivism.

^15^Pavlov himself had only postulated the existence of a second signal system, which was investigated by his students after his death (Joravsky, 1990; Windholz, 1990).

^16^Defectology is the study of mentally handicapped children and adults (Lubovsky, 1974).

^17^A complete bibliography of Luria’s published works in Russian, English, and other languages can be found in Homskaya (2001).

^18^Anatoli Ivanov Smolensky (1895–1982), Director of Highest Nervous Activity of the Academy of Sciences; A. Asratyan (1903–1981), Chief of Physiology of the Academy of Sciences; and K. M. Bykov (1886–1959), Director of The Pavlov Institute of Physiology.

^19^Petr Kupalov (1888–1964), Director of the Pavlov Laboratory, and Leon Orbeli, Director of the Laboratory of Evolutionary Physiology.

^20^Penfield (1955, 896) in his report on his visit to Pavlov’s laboratory.

^21^Like Babkin, Anrep was a distinguished student of Pavlov, and like Babkin he took refuge in Starling’s laboratory at University College London, in Anrep’s case after fighting against the Bolsheviks in the Russian Civil War (Yentis, 1998).

^22^Roberts benefited from the advice of Francis Schiller, Pierre Paul Broca’s biographer, who had come to the Montreal Neurological Institute as a special research fellow shortly after publishing *A study on Aphasia studied in patients with missile wounds* (Schiller, 1947, 1979).

^23^Penfield (1949a,b) first described the language function of the supplementary motor area and its integration with Broca’s and Wernicke’s areas in 1949.

^24^Charcot and his student Pitres were the first to localize the motor representation of the hand to the mid-aspect of the precentral gyrus (Leblanc, 2021).
